# Macrofilaricidal Activity in *Wuchereria bancrofti* after 2 Weeks Treatment with a Combination of Rifampicin plus Doxycycline

**DOI:** 10.1155/2011/201617

**Published:** 2011-05-22

**Authors:** Alexander Yaw Debrah, Sabine Mand, Yeboah Marfo-Debrekyei, Linda Batsa, Anna Albers, Sabine Specht, Ute Klarmann, Kenneth Pfarr, Ohene Adjei, Achim Hoerauf

**Affiliations:** ^1^Faculty of Allied Health Sciences, Kwame Nkrumah University of Science and Technology, UPO, PMB, Kumasi, Ghana; ^2^Kumasi Centre for Collaborative Research in Tropical Medicine (KCCR), Kwame Nkrumah University of Science and Technology, UPO, PMB, Kumasi, Ghana; ^3^Institute for Medical Microbiology, Immunology and Parasitology, University Clinic Bonn, Sigmund-Freud-Stra*β*e 25, D-53105 Bonn, Germany; ^4^School of Medical Sciences, Kwame Nkrumah University of Science and Technology, UPO, PMB, Kumasi, Ghana

## Abstract

Infection with the filarial nematode *Wuchereria bancrofti* can lead to lymphedema, hydrocele, and elephantiasis. Since adult worms cause pathology in lymphatic filariasis (LF), it is imperative to discover macrofilaricidal drugs for the treatment of the infection. Endosymbiotic *Wolbachia* in filariae have emerged as a new target for antibiotics which can lead to macrofilaricidal effects. In Ghana, a pilot study was carried out with 39 LF-infected men; 12 were treated with 200 mg doxycycline/day for 4 weeks, 16 were treated with a combination of 200 mg doxycycline/day + 10 mg/kg/day rifampicin for 2 weeks, and 11 patients received placebo. Patients were monitored for *Wolbachia* and microfilaria loads, antigenaemia, and filarial dance sign (FDS). Both 4-week doxycycline and the 2-week combination treatment reduced *Wolbachia* load significantly. At 18 months posttreatment, four-week doxycycline resulted in 100% adult worm loss, and the 2-week combination treatment resulted in a 50% adult worm loss. In conclusion, this pilot study with a combination of 2-week doxycycline and rifampicin demonstrates moderate macrofilaricidal activity against *W. bancrofti*.

## 1. Introduction

Approximately 120 million people in Africa, Asia, and America are infected with the filarial nematodes Wuchereria bancrofti or Brugia malayi, resulting in lymphatic filariasis (LF), of which more than 40 million suffer from the debilitating lymphatic pathologies such as lymphedema, elephantiasis, and hydrocele. LF is one of the most common causes of global disability [1]. The classical drugs diethylcarbamazine (DEC), ivermectin (IVM), and albendazole (ALB) have been used for the last two decades as the major mode of intervention for filarial infection in successful mass drug administration (MDA) programmes [2–4]. However, these drugs are mainly microfilaricidal [5–10], that is, killing the first larval stage, the microfilariae (Mf). The currently available regimes have been optimised to obtain a decrease in transmission by reducing the Mf load in a given population at both low cost and logistical effort. 

Because ivermectin does not, and DEC plus albendazole only partially kill adult worms that are the causes of pathology in LF (urogenital disorders and lymphedema), research is ongoing to discover novel antifilarial drugs with strong macrofilaricidal activity. A macrofilaricidal drug would help to prevent excruciating sequela and ensure elimination of the disease as a public health problem. One promising area is the use of antibiotics to deplete the *Wolbachia* endosymbionts found in most filarial species, which leads to the inhibition of worm development, embryogenesis, fertility, and viability in human onchocerciasis [11–14] and LF [15–18]. We have recently demonstrated that a four-week course of doxycycline, followed by a single dose of ivermectin treatment in bancroftian filariasis, is effective at reducing *Wolbachia *levels by 95% and, more importantly, it leads to strong macrofilaricidal activity with improvement of pathology [16, 17]. However, a shorter regimen with antibiotics or more effective antifilarial drugs would be advantageous for the treatment of the large populations or for individual treatment, for example, in outpatient clinics, and for children.

A three-week course of doxycycline followed by a single dose of ivermectin/albendazole treatment had been shown by us to reduce *Wolbachia* single gene copies per microfilaria (Mf) by 86%, which abolished microfilaraemia for more than one year and ameliorated adverse reactions to standard antifilarial drugs used for mass chemotherapy [19]. However, this treatment did not show macrofilaricidal activity. Nevertheless, three-week doxycycline in combination with a single dose of DEC did show a macrofilaricidal effect [20], demonstrating that different combinations with doxycycline could lead to a shorter regimen with macrofilaricidal effect. 

Studies carried out by different research groups, both *in vitro* and *in vivo* in animal models, indicate that, besides doxycycline, other antibiotics registered for human use, especially rifampicin, also reduced *Wolbachia* from adult worms and damaged developing embryos and, at a higher concentration, killed adult worms [21–23]. In fact, in murine filariasis using *Litomosoides sigmodontis*, administration of rifampicin in combination with doxycycline could reduce the treatment time by approximately 50% [23, Specht and Hoerauf, unpublished]. This implies that a combination of rifampicin and doxycycline might be complementary to each other leading to a macrofilaricidal activity with a shorter effective regimen and a better *Wolbachia* depletion capacity. Rifampicin has also been tried in human onchocerciasis and 2–4 weeks of 10 mg/kg/d showed antiwolbachial activity and reduction of worm fertility [24]. Based on these promising results from animal and human trials, and given that rifampicin is a registered drug that can be taken by children, clinical trials were initiated to investigate its antifilarial properties against LF in humans.

Thus, the intention of this trial was to test if the combination of doxycycline and rifampicin would be more effective against bancroftian filariasis in humans compared to doxycycline alone. In this study, we report on a trial with doxycycline (200 mg/day) alone for 4 weeks versus a combination of rifampicin (10 mg/kg body weight/day) plus doxycycline (200 mg/day) for two weeks versus placebo matching doxycycline.

## 2. Materials and Methods

This randomized open trial was conducted in the coastal villages of Asanta, Sanwoma, Agyambra, and Miamia in the Nzema East and the Ahanta West District in the Western Region of Ghana from 2005 to 2008. The study site was selected based on an established occurrence of *W. bancrofti* infection within the surrounding region and clinical observations consistent with symptomatic disease in the villagers [16, 25, 26]. Written informed consent was obtained from all participants, and the study was approved by the Committee on Human Research, Publication and Ethics of the Kwame Nkrumah University of Science and Technology and Komfo Anokye Teaching Hospital, Kumasi, Ghana. The study conformed to the principles of the Helsinki Declaration of 1964 (as revised in 1983, 2000, and 2004).

Individuals eligible for participation were adult men aged 18–60 years, with a minimum body weight of 40 kg, in good health without any clinical condition requiring chronic medication. Exclusion criteria encompassed a microfilarial load <20 Mf/mL, abnormal hepatic and renal enzymes (above AST [0–40 IU/L], ALT [0–45 IU/L], creatinine [53–126 *μ*mol/L]) assessed by dipstick chemistry, and alcohol or drug abuse. Leukocyte and differential counts of thin blood smears were also done.

### 2.1. Doxycycline and Rifampicin Treatment

**Figure 1:**
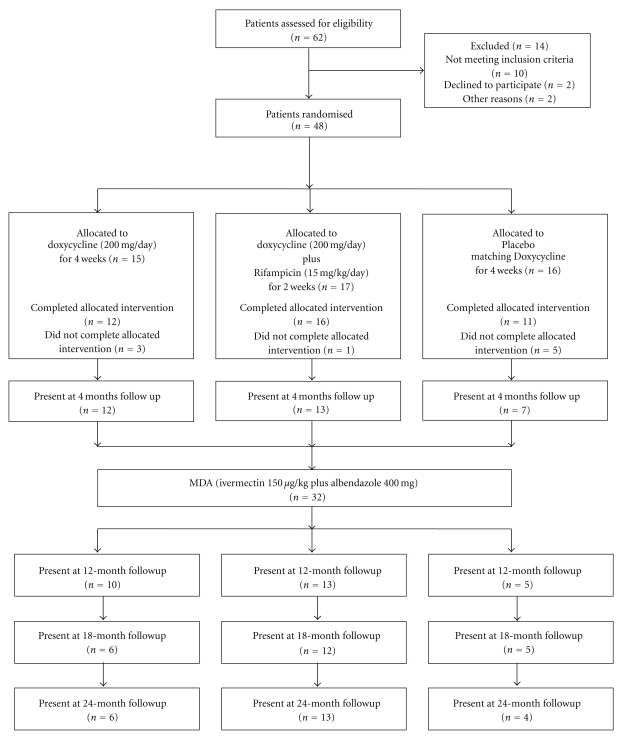
Recruitment and treatment profile of the pilot study.

In all, 48 men were recruited for the study, and 39 completed the treatment course (Figure 1). Nine men dropped out and could not complete the treatment because they either moved from the villages or could not comply with the daily observed treatment because of fishing activities. Of the 39 men, 12 were treated with 200 mg doxycycline/d for 4 weeks, 16 patients with 200 mg doxycycline/d + 10 mg/kg/d rifampicin for 2 weeks + 2 weeks placebo matching doxycycline, and 11 patients were treated with placebo matching doxycycline for 4 weeks. Due to unavailability of rifampicin placebo at the time, the rifampicin plus doxycycline treatment arm was an open study while for the doxycycline capsules matching placebo were available so this part of the trial was placebo controlled. 

Treatment was administered and monitored by a trial clinician on a daily basis. Four months after the start of treatment, all participants received a standard oral dose of 400 mg albendazole (GlaxoSmithkline) and 150 *μ*g/kg ivermectin (Mectizan, Merck, Sharp & Dohme) in 2006 and received another dose at the end of the treatment in 2008.

### 2.2. Outcome Measurement

The primary outcome measurement of this study was assessment of adult worm vitality using ultrasonography (USG) [26].

### 2.3. Ultrasound (USG) Examinations

Study participants were examined before treatment and at 12, 18, and 24 months using a portable ultrasound machine (SONOSITE 180 Plus) equipped with a 7.5 MHZ linear transducer as previously described [26, 27]. Patients were screened for living adult filarial worms detectable in lymphatic vessels of the scrotum and the spermatic cord. Ultrasound findings were documented by printouts and digital video recordings of each worm nest in the b-, m- and Pulse Wave Doppler-modes. The locations of the worm nest(s) (e.g., left or right supra-, para- and infratesticular area, lymphatic vessels of the spermatic cord) were documented for longitudinal analysis of appearance, stability, or disappearance, respectively.

### 2.4. Determination of Microfilarial (Mf) Load

For a quick screening at night to identify people who had Mf, the *W. bancrofti* Mf load was determined by microscopic examination of finger prick blood samples [25] in the communities. Subsequently, eligible patients donated 10 ml of venous blood for accurate quantification using Whatman Nucleopore filter method as described previously [25]. The same volume of blood was taken from patients present at 4, 12, 18, and 24 months after the commencement of treatment. At each time point, plasma was taken from the remaining sample, aliquoted, and frozen at −80°C for later analysis of antigenaemia.

### 2.5. Determination of Wolbachia Levels in Mf by PCR

*Wolbachia *content was quantified by real-time PCR of the *W. bancrofti Wolbachia*–*ftsZ* gene (AF081198 NCBI) derived from 500–1000 microfilariae using a RotorGene 3000 (Corbett Research, Inc., Sydney, Australia) at pretreatment and 4 months after treatment onset. Details of the technique are given elsewhere [16, 25]. Briefly, DNA was extracted from 500–1000 Mf trapped on a Whatman filter using the QIAamp kit (Qiagen, Hilden, Germany) following the kit protocol with the exception that the 56°C incubation was performed overnight. As previously described [16, 25], primers amplified a 256 bp fragment of the *Wolbachia*-*ftsZ* gene, and a TaqMan probe (Invitrogen, Darmstadt, Germany) for the same sequence was used to detect the product by real-time PCR. The PCR products were quantified by comparing with a standard curve of the plasmid containing the *ftsZ* fragment.

### 2.6. Determination of Circulating Filarial Antigenaemia

Adult *W. bancrofti *antigen was measured with the TropBio ELISA test kit (TropBio, Townsville, Australia). The kit protocol was followed with the following exceptions: samples were boiled in sample diluent and diluted (1 : 20 ratio) with the diluent [14, 15]; 50 *μ*L of the diluted plasma was added to plate wells; the plates were incubated overnight. Samples were tested in duplicate. The optical density at 414 nm was recorded from plasma samples taken before treatment and from the 12-, 18-, and 24-month followup time points. Antigen units were determined using a standard curve from standards provided by the manufacturer and the final units multiplied by the dilution factor of 20.

### 2.7. Data Analysis

Depletion of *Wolbachia *from Mf, the number of microfilaria and filarial dance sign (FDS) and the levels of circulating filarial antigen (CFA) were summarized as medians and 25th and 75th percentiles for the different treatment groups. Changes in medians at baseline and subsequent followups were analyzed using Wilcoxon Signed Rank test. The Kruskal-Wallis test and the Mann-Whitney-U test were used for comparisons between the treatment groups. 

Mf-positive individuals as well as individuals showing filarial dance sign (FDS) before and after treatment were given as number of positive individuals per total number of persons examined and as percentages. The differences between the treatment groups were evaluated using Fisher's exact test. Two-tailed *P* values lower than  .05 were considered significant. 

All analyses were done using Predictive Analysis Software (PASW) version 18.

## 3. Results

Forty-eight men were randomized for the study, and 39 completed the treatment (Figure 1). Of the 39 men, 12 were treated with 200 mg doxycycline/day for 4 weeks, 16 were treated with a combination of 200 mg doxycycline/day + 10 mg/kg/day rifampicin for 2 weeks, and 11 patients received placebo matching doxycycline. Nine patients did not complete the allocated intervention, three from the doxycycline 4-week arm, one from the doxycycline + rifampicin combination, and five from the placebo group. There was no statistical difference among the three groups regarding the age distribution (*P* = .287, Kruskal-Wallis test).

### 3.1. Adverse Events Experienced by Patients during Treatment

All drugs were well tolerated. In all, eight out of the 48 patients randomized for the treatment experienced slight adverse effects. Three patients were from the placebo group, another three from the 200 mg doxycycline/d + 10 mg/kg/d rifampicin group, and two were from the 4-week doxycycline group. Of the three placebo-treated patients, one had diarrhea for one day, and the two had dizziness for one day and three days, respectively. Of the three rifampicin- and doxycycline-treated group, one had nausea after eating for one day and two experienced dizziness for a day each; from the 4-week doxycycline group, one had stomachache for a day and another had headache with cough for 3 days. Oral rehydration salt was administered to the patients with gastrointestinal complaints. Apart from this, no interventions were needed. There was no statistical difference in the number of patients who experienced adverse events between the three groups (*P* = .847).

### 3.2. Primary Outcome Analysis

#### 3.2.1. Measurement of Adult Worm Vitality

Since ultrasonography is noninvasive and has been proven to be effective for assessing a macrofilaricidal effect [16, 17, 27–29], this method was used as the primary tool for this study, while circulating filarial antigen (CFA) levels in plasma were assessed as a confirmative parameter. At 12 months, the 4-week doxycycline group had 80% worm loss while the combination treatment of 2-week doxycycline + rifampicin had 38% worm loss and the placebo group had no change in worm vitality (Table 1). The same trend could be seen in the number of FDS. While there was a decrease of FDS in the doxycycline and the combination group (*P* = .006 and *P* = .034, resp.; Wilcoxon signed rank test), there was rather an increase in the placebo group (*P* = .083, Table 1). At 18 months after treatment, this pattern remained the same with the 4-week doxycycline group having 100% loss of worm nests, the 2-week doxycycline + rifampicin group showing a 50% loss of worm nests while placebo group remained unchanged (Table 1). This trend was also observed at 24 months but less significantly (Table 1), which is most likely due to possible reinfection of the patients, as observed earlier [16]. There was not significant reduction in the antigenaemia compared to the loss of worms by USG as shown in our previous studies [14–18]. This was probably due to very few people donating blood during the followup time points for the antigenaemia analyses.

### 3.3. Secondary Outcomes

Table 1 also illustrates the changes in microfilaraemia and *Wolbachia/*Mf from baseline and at followup time points. Baseline measurements were similar between the allocated treatment groups, except the Mf load which was higher in the placebo group than the two treatment groups (*P* = .001, Kruskal-Wallis test). However, the Mf loads between the 4-week doxycycline group and the 2-week doxycycline + rifampicin group were comparable (*P* = .378, Mann-Whitney-U test) (Table 1). At 12 months after treatment, the 4-week doxycycline regimen completely reduced the Mf load (*P* = .012). Significant reductions in microfilaraemia were also seen in the 2-week doxycycline + rifampicin (*P* = .003) and the placebo (*P* = .043) regimens (Table 1). However, at 18 and 24 months, while the 4-week doxycycline and 2-week doxycycline + rifampicin regimens continued to maintain significantly low microfilarial loads, there was a rise in Mf loads in the placebo patients (Table 1).

The *Wolbachia* loads in the Mf (*ftsZ *copies*/*Mf) at four months posttreatment were reduced by 93% (*P* = .002, Wilcoxon signed rank test) in the 4-week doxycycline group, and 85% (*P* = .034, Wilcoxon signed rank test) in the 2-week doxycycline + rifampicin group, but there was no change in the placebo group (*P* = .138, Wilcoxon signed rank test, Table 1).

## 4. Discussion

Lymphatic filariasis (LF) is a common cause of disability in humans. Of the presently used antifilarial drugs to combat LF within the scope of MDA only DEC—which is not used in African countries coendemic for onchocerciasis—has moderate macrofilaricidal activity [30–33]. Doxycycline is a safe and efficacious drug for treatment of LF infection. Previous trials indicate that an eight-, a six- or a four-week course of 200 mg/day followed by a single dose of ivermectin or a three-week course of 200 mg/day followed by a single dose of DEC leads to gradual and sustained loss of Mf from host blood and has strong macrofilaricidal activity [15–18, 34, 35]. The advantage of a macrofilaricidal drug is that it targets the adult worms that are the inducers of lymphatic pathology and thus can bring direct relief to individual patients that seek treatment, for example, in outpatient clinics, without having to rely on the indirect means of reduction of infection pressure due to transmission control by microfilaricidal drugs [36]. However, the previously published doxycycline regimens are long, and a shorter regimen is desirable to complement classical antifilarial drugs [36]. 

The present trial confirmed a previous report that 4-week doxycycline results in more than 80% macrofilaricidal effect [17]. The second and novel result of this study is that the combination of rifampicin with doxycycline had an antiwolbachial effect on *W. bancrofti* in humans and this effect occurred with a shorter (2 weeks) course of treatment. Here we describe the maintenance of very low microfilaraemia at 12 and 18 months after doxycycline + rifampicin treatment and a moderate (50%) macrofilaricidal activity indicated by the loss of worm nests in the combination group. In contrast, in the placebo patients the worm nests remained stable throughout the followup examinations. 

As discussed above, our three-week course of doxycycline alone was shown to abolish microfilaraemia for more than one year [19]; however, this treatment did not show macrofilaricidal activity. Nevertheless, the same three-week doxycycline regimen with DEC did show a macrofilaricidal effect [20], demonstrating that the DEC might have some added effect on doxycycline. Our current results show that a treatment with a combination of doxycycline and rifampicin for two weeks also has a macrofilaricidal effect. Thus, this pilot study has provided indirect evidence that rifampicin might have a synergetic effect with doxycycline. A current field try aims to confirm the synergetic effects of these two drugs. 

It is likely that 2-week doxycycline + rifampicin could have stronger macrofilaricidal activity than the 50% observed when patients are observed after a longer period. However, due to the high transmission rate in this area, longer followups are not useful because of reinfection. Notwithstanding, the present study showing 2-week doxycycline + rifampicin having a partial macrofilaricidal effect is encouraging in the sense that it serves as further proof that antiwolbachial therapy as a treatment against LF may be possible with a shorter regimen if antiwolbachial drugs are combined. 

The results of the combination of 3-week doxycycline and rifampicin in an ongoing trial will provide more information.

##  Coflict of Interests

The authors declare no conflict of interests.

##  Authors' Contribution 

Alexander Yaw Debrah participated in patient recruitment, performed the laboratory (Mf, *Wolbachia*, and CFA) analyses, compiled the data, and drafted the manuscript. Sabine Mand participated in patient recruitment, blood taking, and doxycycline daily treatment and performed the ultrasonography and the followup examinations. Yeboah Marfo-Debrekyei participated in the negotiations with the village elders, patient recruitment, and doxycycline daily treatment. Linda Batsa participated in patient recruitment, performed the laboratory analysis (Mf, *Wolbachia*, and CFA) and helped in compiling the data. Anna Albers participated in the laboratory (*Wolbachia*) analysis. Ute Klarmann did the statistical analysis. Sabine Specht participated in the laboratory (CFA) analysis. Kenneth Pfarr participated in the laboratory (*Wolbachia*) analysis. Ohene Adjei interacted with the local authorities (ethics committees, district health, etc.) and supervised the local data collection. Achim Hoerauf conceived the idea, designed, and supervised the study and edited the final manuscript version.

## Figures and Tables

**Table 1:** Variables of the patients who completed the treatment.

	Pretreatment	4 months	Pretreatment	12 months	Pretreatment	18 months	Pretreatment	24 months
**Doxycycline 4 weeks**								

MF-positive individuals/all (%)	12/12 (100%)	9/12 (75%)	8/8 (100%)	0/8 (0%)	5/5 (100%)	0/5 (0%)	6/6 (100%)	3/6 (50%)
*Comparison to Rifampicin plus Doxycycline* ^§^		*P* = 1.0		*P* = .007		*P* = .044		*P* = .262
*Comparison to Placebo* ^§^		*P* = .515		*P* = .001		*P* = .008		*P* = .2

Microfilaraemia								
Median (25th–75th percentile)	523 (154–1013)	307 (12–1068)	523 (227–1006)	0	1100 (372–2010)	0	667 (193–838)	12 (0–49)
Comparison between time points*		*P* = .388		*P* = .012		*P* = .043		*P* = .028
*Comparison to Rifampicin plus Doxycycline* ^#^	*P* = .378	*P* = .954		*P* = .008		*P* = .039		*P* = .25
*Comparison to Placebo* ^#^	*P* = .007	*P* = .072		*P* = .001		*P* = .007		*P* = .018

*Wolbachia* load/Mf								
Median (25th–75th percentile)	167 (71–238)	11 (4–24)						
Comparison between time points*		*P* = .002						
*Comparison to Rifampicin plus Doxycycline* ^#^	*P* = .626	*P* = .326						
*Comparison to Placebo* ^#^	*P* = .31	*P* = .003						

Antigenaemia								
Median (25th–75th percentile)	14500 (9421–19162)	20574 (18369–29241)	16459 (10141–17843)	22854 (11341–17784)	11341 (9108–16776)	17625 (9711–118594)	13840 (9901–18283)	28065 (15442–49163)
Comparison between time points*		*P* = .158		*P* = .237		*P* = .173		*P* = .075
*Comparison to Rifampicin plus Doxycycline* ^#^	*P* = .71	*P* = .828		*P* = .362		*P* = 1.0		*P* = .512
*Comparison to Placebo* ^#^	*P* = .14	*P* = .8		*P* = .57		*P* = .302		*P* = 1.0

FDS-positive individuals/all (%)			10/10 (100%)	2/10 (20%)	5/5 (100%)	0/5 (0%)	4/4 (100%)	1/4 (25%)
*Comparison to Rifampicin plus Doxycycline* ^§^			*P* = .429	*P* = .09		*P* = .101		*P* = .569
*Comparison to Placebo* ^§^			*P* = .317	*P* = .007		*P* = .008		*P* = .143

No. of FDS								
Median (25th–75th percentile)			1.0 (1–3.25)	0 (0–0.25)	2.0 (0.5–5)	0	1.0 (0.25–1.75)	0
Comparison between time points*				*P* = .006		*P* = .068		*P* = .564
*Comparison to Rifampicin plus Doxycycline* ^#^			*P* = .796	*P* = .081		*P* = .065		*P* = .555
*Comparison to Placebo* ^#^			*P* = .824	*P* = .003		*P* = .006		*P* = .178

**Rifampicin plus Doxycycline 2 weeks**								

MF-positive individuals/all (%)	12/12 (100%)	10/12 (83%)	13/13 (100%)	8/13 (62%)	12/12 (100%)	7/12 (58%)	13/13 (100%)	11/13 (85%)
*Comparison to Placebo* ^$^		*P* = 1.0		*P* = .249		*P* = .245		*P* = 1.0

Microfilaraemia								
Median (25th–75th percentile)	392 (122–460)	394 (39–703)	413 (109–465)	26 (0–133)	392 (183–468)	13 (0–64)	371 (130–465)	17 (1–290)
Comparison between time points*		*P* = .695		*P* = .003		*P* = .002		*P* = .004
*Comparison to Placebo* ^#^	*P* < .001	*P* = .058		*P* = .015		*P* = .019		*P* = .031

*Wolbachia* load/Mf								
Median (25th–75th percentile)	121 (95–200)	18 (7–52)						
Comparison between time points*		*P* = .034						
*Comparison to Placebo* ^#^	*P* = .537	*P* = .015						

Antigenaemia								
Median (25th–75th percentile)	15379 (10154–21680)	23193 (19514–29162)	15942 (10356–21680)	19928 (17305–22719)	13063 (10186–21852)	19480 (6432–28276)	17095 (10247–22273)	41933 (18834–51020)
Comparison between time points*		*P* = .046		*P* = .196		*P* = .508		*P* = .015
*Comparison to Placebo* ^#^	*P* = .084	*P* = .606		*P* = .034		*P* = .091		*P* = .808

FDS-positive individuals/all (%)			13/13 (100%)	8/13 (62%)	10/10 (100%)	5/10 (50%)	12/12 (100%)	7/12 (58%)
*Comparison to Placebo* ^§^			*P* = .056	*P* = .249		*P* = .221		*P* = .245

No. of FDS								
Median (25th–75th percentile)			1.0 (1–1.5)	1.0 (0–1)	1.0 (1–2)	0.5 (0–1)	1.0 (1–2)	1.0 (0–2)
Comparison between time points*				*P* = .034		*P* = .011		*P* = .014
*Comparison to Placebo* ^#^			*P* = .936	*P* = .019		*P* = .103		*P* = .162

**Placebo matching Doxycycline 4 weeks**								

MF-positive individuals/all (%)	5/5 (100%)	5/5 (100%)	5/5 (100%)	5/5 (100%)	4/4 (100%)	4/4 (100%)	4/4 (100%)	4/4 (100%)

Microfilaraemia								
Median (25th–75th percentile)	2380 (1230–3680)	2585 (412–3738)	2810 (1970–3820)	283 (105–358)	1595 (513–4008)	451 (82–1910)	1790 (503–4115)	605 (164–1194)
*Comparison between time *points*		*P* = .686		*P* = .043		*P* = .273		*P* = .273

*Wolbachia* load/Mf								
Median (25th–75th percentile)	133 (85–164)	358 (100–879)						
Comparison between time points*		*0.138*						

Antigenaemia								
Median (25th–75th percentile)	12616 (10398–97319)	21861 (18614–26987)	12616 (10345–58134)	27747 (26039–34228)	97319	28653	48995 (10318–98789)	30902 (7541–76947)
Comparison between time points*		*P* = .866		*P* = .5		*P* = .285		*P* = .465

FDS-positive individuals/all (%)			3/5 (60%)	5/5 (100%)	2/4 (50%)	4/4 (100%)	3/4 (75%)	4/4 (100%)

No. of FDS								
Median (25th–75th percentile)			3.0 (0–4)	3.0 (1–4.5)	0.5 (0–2.5)	1.0 (1–1.75)	1.5 (0.25–2.75)	1.5 (1–2.75)
Comparison between time points*				*P* = .083		*P* = .785		*P* = .564

^§^Fisher's exact-test.

*Wilcoxon-signed-rank-test.

^#^Mann-Whitney-U-test.

## Abbreviations

ALB: Albendazole
DEC: Diethylcarbamazine
IVM: Ivermectin
LF: Lymphatic filariasis
MDA: Mass drug administration
Mf: Microfilaria
PCR: Polymerase chain reaction
USG: Ultrasonography.

## References

[1] WHO Annual report on lymphatic filariasis. http://www.filariasis.org/.

[2] WHO (2007). Meeting of the international task force for disease eradication—11 January 2007. *Weekly Epidemiological Record*.

[3] WHO (2007). Global programme to eliminate lymphatic filariasis. Progress report on mass drug administrations in 2006. *Weekly Epidemiological Record*.

[4] WHO (2010). Global programme to eliminate lymphatic filariasis. Progress report on mass drug administrations in 2009. *Weekly Epidemiological Record*.

[5] Awadzi K, Attah SK, Addy ET, Opoku NO, Quartey BT (1999). The effects of high-dose ivermectin regimens on Onchocerca volvulus in onchocerciasis patients. *Transactions of the Royal Society of Tropical Medicine and Hygiene*.

[6] Ottesen EA, Duke BOL, Karam M, Behbehani K (1997). Strategies and tools for the control/elimination of lymphatic filariasis. *Bulletin of the World Health Organization*.

[7] Norões J, Dreyer G, Santos A, Mendes VG, Medeiros Z, Addiss D (1997). Assessment of the efficacy of diethylcarbamazine on adult Wuchereria bancrofti in vivo. *Transactions of the Royal Society of Tropical Medicine and Hygiene*.

[8] Critchley J, Addiss D, Gamble C, Garner P, Gelband H, Ejere H (2005). Albendazole for lymphatic filariasis. *Cochrane Database of Systematic Reviews*.

[9] Gyapong JO, Kumaraswami V, Biswas G, Ottesen EA (2005). Treatment strategies underpinning the global programme to eliminate lymphatic filariasis. *Expert Opinion on Pharmacotherapy*.

[10] Dreyer G, Addiss D, Williamson J, Norões J (2006). Efficacy of co-administered diethylcarbamazine and albendazole against adult Wuchereria bancrofti. *Transactions of the Royal Society of Tropical Medicine and Hygiene*.

[11] Hoerauf A, Volkmann L, Hamelmann C (2000). Endosymbiotic bacteria in worms as targets for a novel chemotherapy in filariasis. *The Lancet*.

[12] Hoerauf A, Mand S, Adjei O, Fleischer B, Büttner DW (2001). Depletion of *Wolbachia* endobacteria in *Onchcerca volvulus* by doxycycline and microfilaridermia after ivermectin treatment. *The Lancet*.

[13] Hoerauf A, Specht S, Marfo-Debrekyei Y (2009). Efficacy of 5-week doxycycline treatment on adult *Onchocerca volvulus*. *Parasitology Research*.

[14] Debrah AY, Mand S, Marfo-Debrekyei Y, Larbi J, Adjei O, Hoerauf A (2006). Assessment of microfilarial loads in the skin of onchocerciasis patients after treatment with different regimens of doxycycline plus ivermectin. *Filaria Journal*.

[15] Taylor MJ, Makunde WH, McGarry HF, Turner JD, Mand S, Hoerauf A (2005). Macrofilaricidal activity after doxycycline treatment of *Wuchereria bancrofti*: a double-blind, randomised placebo-controlled trial. *The Lancet*.

[16] Debrah AY, Mand S, Specht S (2006). Doxycycline reduces plasma VEGF-C/sVEGFR-3 and improves pathology in lymphatic filariasis. *PLoS Pathogens*.

[17] Debrah AY, Mand S, Marfo-Debrekyei Y (2007). Macrofilaricidal effect of 4 weeks of treatment with doxycycline on Wuchereria bancrofti. *Tropical Medicine and International Health*.

[18] Debrah AY, Mand S, Marfo-Debrekyei Y (2009). Reduction in levels of plasma vascular endothelial growth factor-A and improvement in hydrocele patients by targeting endosymbiotic Wolbachia sp. in Wuchereria bancrofti with doxycycline. *American Journal of Tropical Medicine and Hygiene*.

[19] Turner JD, Mand S, Debrah AY (2006). A randomized, double-blind clinical trial of a 3-week course of doxycycline plus albendazole and ivermectin for the treatment of *Wuchereria bancrofti* infection. *Clinical Infectious Diseases*.

[20] Mand S, Pfarr K, Sahoo PK (2009). Macrofilaricidal activity and amelioration of lymphatic pathology in bancroftian filariasis after 3 weeks of doxycycline followed by single-dose diethylcarbamazine. *American Journal of Tropical Medicine and Hygiene*.

[21] Townson S, Hutton D, Siemienska J (2000). Antibiotics and *Wolbachia* in filarial nematodes: antifilarial activity of rifampicin, oxytetracycline and chloramphenicol against *Onchocerca gutturosa*, *Onchocerca lienalis* and *Brugia pahangi*. *Annals of Tropical Medicine and Parasitology*.

[22] Rao R, Weil GJ (2002). In vitro effects of antibiotics on Brugia malayi worm survival and reproduction. *Journal of Parasitology*.

[23] Volkmann L, Fischer K, Taylor M, Hoerauf A (2003). Antibiotic therapy in murine filariasis (*Litomosoides sigmodontis*): comparative effects of doxycycline and rifampicin on *Wolbachia* and filarial viability. *Tropical Medicine and International Health*.

[24] Specht S, Mand S, Marfo-Debrekyei Y (2008). Efficacy of 2- and 4-week rifampicin treatment on the Wolbachia of Onchocerca volvulus. *Parasitology Research*.

[25] Hoerauf A, Mand S, Fischer K (2003). Doxycycline as a novel strategy against bancroftian filariasis-depletion of *Wolbachia* endosymbionts from *Wuchereria bancrofti* and stop of microfiariae production. *Medical Microbiology and Immunology*.

[26] Mand S, Marfo-Debrekyei Y, Dittrich M, Fischer K, Adjei O, Hoerauf A (2003). Animated documentation of the filaria dance sign (FDS) in bancroftian filariasis. *Filaria Journal*.

[27] Dreyer G, Brandão AC, Amaral F, Medeiros Z, Addiss D (1996). Detection by ultrasound of living adult *Wuchereria bancrofti* in the female breast. *Memórias do Instituto Oswaldo Cruz*.

[28] Mand S, Büttner DW, Hoerauf A (2008). Bancroftian filariasis—absence of *Wolbachia* after doxycycline treatment. *American Journal of Tropical Medicine and Hygiene*.

[29] Kshirsagar NA, Gogtay NJ, Garg BS (2004). Safety, tolerability, efficacy and plasma concentrations of diethylcarbamazine and albendazole co-administration in a field study in an area endemic for lymphatic filariasis in India. *Transactions of the Royal Society of Tropical Medicine and Hygiene*.

[30] Norões J, Dreyer G, Santos A, Mendes VG, Medeiros Z, Addiss D (1997). Assessment of the efficacy of diethylcarbamazine on adult Wuchereria bancrofti in vivo. *Transactions of the Royal Society of Tropical Medicine and Hygiene*.

[31] Ottesen EA, Duke BOL, Karam M, Behbehani K (1997). Strategies and tools for the control/elimination of lymphatic filariasis. *Bulletin of the World Health Organization*.

[32] Hussein O, El Setouhy M, Ahmed ES (2004). Duplex Doppler sonographic assessment of the effects of diethylcarbamazine and albendazole therapy on adult filarial worms and adjacent host tissues in bancroftian filariasis. *American Journal of Tropical Medicine and Hygiene*.

[33] Dreyer G, Addiss D, Williamson J, Norões J (2006). Efficacy of co-administered diethylcarbamazine and albendazole against adult *Wuchereria bancrofti*. *Transactions of the Royal Society of Tropical Medicine and Hygiene*.

[34] Hoerauf A (2006). New strategies to combat filariasis. *Expert Review of Anti-Infective Therapy*.

[35] Hoerauf A (2008). Filariasis: new drugs and new opportunities for lymphatic filariasis and onchocerciasis. *Current Opinion in Infectious Diseases*.

[36] Taylor MJ, Hoerauf A, Bockarie M (2010). Lymphatic filariasis and onchocerciasis. *The Lancet*.

